# Conservation of *Erwinia amylovora* pathogenicity-relevant genes among *Erwinia* genomes

**DOI:** 10.1007/s00203-017-1409-7

**Published:** 2017-07-10

**Authors:** Luigimaria Borruso, Marco Salomone-Stagni, Ivan Polsinelli, Armin Otto Schmitt, Stefano Benini

**Affiliations:** 1 Bioorganic Chemistry and Bio-Crystallography Laboratory (B2Cl), Faculty of Science and Technology, Free University of Bolzano, Piazza Università 5, 39100 Bolzano, Italy; 2 Department of Nutztierwissenschaften, Breeding Informatics, Georg-August-Universität Göttingen, Carl-Sprengel-Weg 1, 37075 Göttingen, Germany

**Keywords:** Fire blight, Comparative genomics, BLAST, Pathogenesis, Virulence factor

## Abstract

The *Erwinia* genus comprises species that are plant pathogens, non-pathogen, epiphytes, and opportunistic human pathogens. Within the genus, *Erwinia amylovora* ranks among the top 10 plant pathogenic bacteria. It causes the fire blight disease and is a global threat to commercial apple and pear production. We analyzed the presence/absence of the *E. amylovora* genes reported to be important for pathogenicity towards Rosaceae within various *Erwinia* strains genomes. This simple bottom-up approach, allowed us to correlate the analyzed genes to pathogenicity, host specificity, and make useful considerations to drive targeted studies.

## Introduction

*Erwinia amylovora* is a Gram negative bacterium affiliated to the Enterobacteriaceae family and the first phytopathogenic bacterium ever described (Vanneste 2000). *E. amylovora* is the aetiological agent of the fire blight disease in Rosaceae and represents a major global threat to commercial apple and pear production (Norelli et al. 2003; Van der Zwet et al. 2012; Vanneste 2000). A fire blight outbreak may cause the loss of the entire annual harvest and lead to a dramatic economic damage (e.g., in the year 2000 Michigan economy lost $42 million) (Norelli et al. 2003). Weather condition markedly influence *E. amylovora* growth. Therefore, disease-forecasting models have been developed to prevent the disease onset by spraying chemicals when the weather conditions are predicted favorable to *E. amylovora* proliferation (Shtienberg et al. 2003; Van der Zwet et al. 1994). The infection usually occurs in spring when the temperature increases over 18 °C and it spreads by both insects and rain. The disease starts when the bacteria infect the plant through the flower nectarthodes, or through wounds. Within a few days, the infection diffuse rapidly to the whole blossom and young shoots. In a few months, the disease spreads to the whole plant becoming systemic (Smits et al. 2013; Vanneste 2000). Typical symptoms include flower necrosis, blighted shoots and woody tissues cankers. Besides, a common sign of fire blight is the appearance of bacterial ooze. Currently, the main methods to control fire blight are quarantine, pruning and/or eradication of the plants, the use of biological and chemical pesticides, antibiotics and resistant cultivars obtained by classical breeding, or by genetic engineering (Gusberti et al. 2015). However, antibiotics and genetically modified plants are not allowed in most countries where prevention of infections is still the main control method. Several studies upon *E. amylovora* physiology and genetics have shed light on its pathogenicity at the molecular level, bringing out the major virulence factors (Piqué et al. 2015; Smits et al. 2011). Aiming to a better understanding of the gene-pathogenicity and gene–host relationships, we have selected the DNA sequences encoding proteins that are reported to be important in the pathogenesis of *E. amylovora* and we investigated their presence/absence within the strains of *Erwinia* whose genomes are sequenced and assembled (Ancona et al. 2013, 2015, 2016; Bereswill and Geider 1997; Coyne et al. 2013; Du and Geider 2002; Edmunds et al. 2013; Kube et al. 2008, 2010; Mann et al. 2013; Nissinen et al. 2007; Oh and Beer 2005; Pester et al. 2012; Piqué et al. 2015, Smits et al. 2011; Wang et al. 2009, 2011; Zeng et al. 2013; Zhao and Qi 2011).

## Material and methods

**Fig. 1:**
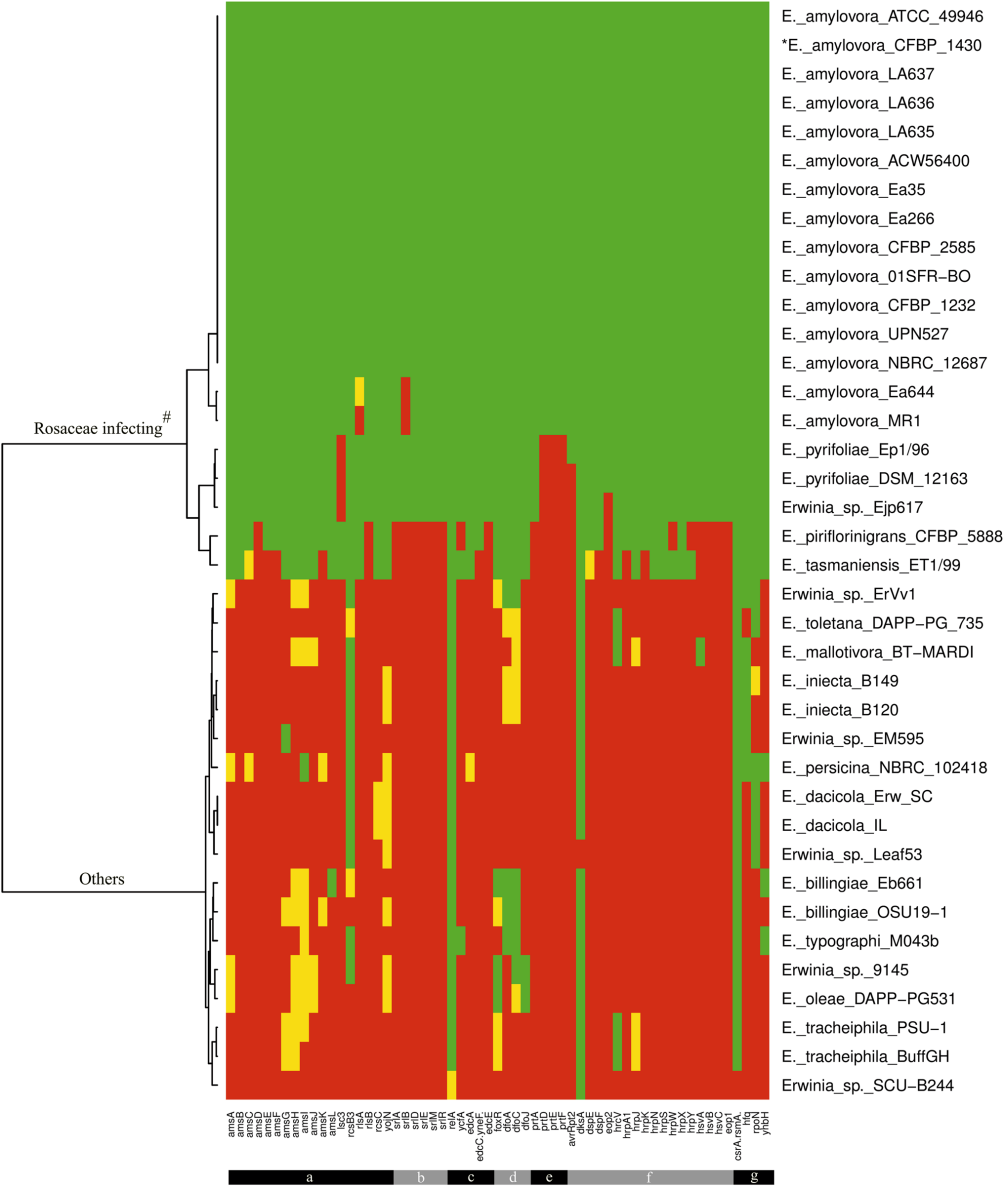
Heatmap hierarchical clustering: on the *right*, *Erwinia* strains are listed; on the bottom, genes important for virulence within *E. amylovora* are reported. Color code: *green* indicates a coverage ≥80% with an identity between 100% and 80%, *yellow* indicates a coverage ≥80% with an identity lower than 75%, red indicates a coverage lower than 75% that is interpreted as the absence of the paralogue. The genes are grouped according to the functional system: **a** exopolysaccharide metabolism, **b** sorbitol metabolism, **c** guanine derivative regulation, **d** desferrioxamine pathway, **e** type 1 secretion system, **f** type 3 secretion system, **g** others (transcription regulator, two-component transduction, positive regulator of virulence factor and sRNA chaperone). ^#^These strains are Rosaceae-infecting apart from *E. tasmaniensis* ET1/99. The figure was rendered with the Krita software

The DNA sequences of 59 genes belonging to *Erwinia amylovora* CFBP1430 (reference genome) and encoding proteins, reported to be important for pathogenicity in *E. amylovora,* were extracted from the European Nucleotide Archive (ENA; http://www.ebi.ac.uk/ena). The genomes of the 38 *Erwinia* strains analyzed in this study derived from the NCBI-Genome database (Table 1). The 59 DNA sequences were BLASTed against the 38 *Erwinia* genomes DNA via the command-line annotation tool Blast, using default settings. A Heatmap Hierarchical Clustering based on Euclidean Distance method was generated via the R software using the heatmap() function from the R Base Package (Fig. 1) (R Core Team 2012). The identity threshold was set according to the following criteria: (1) DNA sequences with a coverage ≥80% and identity ≥75% were marked in green. (2) Sequences with a coverage ≥80% and identity <75% are marked in yellow. (3) Sequences with a coverage <75% were interpreted as the absence of the paralogue and are marked in red. The sequences were grouped according to the following functional systems: exopolysaccharide metabolism, type 3 secretion system (T3SS), positive regulator of virulence factor, desferrioxamine pathway, guanine derivative regulation, sRNA chaperone, two-component signal transduction system, type 1 secretion system, transcription regulator, sorbitol metabolism.

**Table 1:** Characteristic of the *Erwinia* genome strains analyzed in this study

Strain	Accession number	Habitat/host	Plant pathogenicity
*E. amylovora* ATCC 49946^#^	GCA_000027205.1	*Malus* sp. (apple tree)	Pathogen of Spiraeoideae^a^ (Mann et al. 2013)
*E. amylovora* CFBP1430^*,#^	GCA_000091565.1	*Crataegus* (hawthorn)	Pathogen of Spiraeoideae (Mann et al. 2013)
*E. amylovora* LA637^#^	GCA_000513355.1	*Malus* sp. (apple tree)	Pathogen of *Malus* sp. (Smits et al. 2014)
*E. amylovora* LA636^#^	GCA_000513395.1	*Malus* sp. (apple tree)	Pathogen of *Malus* sp. (Smits et al. 2014)
*E. amylovora* LA635^#^	GCF_000513415.1	*Malus* sp. (apple tree)	Pathogen of *Malus* sp. (Smits et al. 2014)
*E. amylovora* ACW56400^#^	GCF_000240705.2	*Pyrus communis* (pear tree)	Pathogen of Spiraeoideae (Mann et al. 2013)
*E. amylovora* Ea356^#^	GCF_000367545.1	*Cotoneaster* sp. (garden shrubs)	Pathogen of Spiraeoideae (Mann et al. 2013)
*E. amylovora* Ea266^#^	GCA_000367565.2	*Malus* sp. (apple tree)	Pathogen of Spiraeoideae (Mann et al. 2013)
*E. amylovora* CFBP 2585^#^	GCF_000367585.2	*Sorbus* sp. (rowan)	Pathogen of Spiraeoideae (Mann et al. 2013)
*E. amylovora* 01SFR BO^#^	GCA_000367605.2	*Sorbus* sp. (rowan)	Pathogen of Spiraeoideae (Mann et al. 2013)
*E. amylovora* CFBP 1232^#^	GCA_000367625.2	*Pyrus communis* (pear tree)	Pathogen of Spiraeoideae (Mann et al. 2013)
*E. amylovora* UPN527^#^	GCA_000367645.1	*Malus* sp. (apple tree)	Pathogen of Spiraeoideae (Mann et al. 2013)
*E. amylovora* NBRC 12687^b,#^	GCA_000696075.1	*Pyrus communis* (pear tree)^c^	–
*E. amylovora* Ea644^#^	GCA_000696075.1	*Rubus idaeus* (raspberry)	Pathogen of *Rubus* (Mann et al. 2013)
*E. amylovora* MR1^#^	GCA_000367685.2	*Rubus idaeus* (raspberry)	Pathogen of *Rubus* (Mann et al. 2013)
*E. pyrifoliae* Ep1/96^#^	GCA_000027265.1	*Pyrus pyrofolia* (asian pear tree/nashi)	Pathogen of *Pyrus pyrifolia* (Kube et al. 2010)
*E. pyrifoliae* DSM-12163^#^	GCA_000026985.1	*Pyrus pyrifolia* (asian pear tree/nashi)	Pathogen of *Pyrus pyrifolia* (Geider et al. 2009)
*Erwinia* sp. Ejp617^#^	GCA_000165815.1	*Pyrus pyrifolia* (asian pear tree/nashi)	Pathogen of *Pyrus pyrifolia* (Park et al. 2011)
*E. piriflorinigrans* CFBP-5888^#^	GCA_001050515.1	*Pyrus communis* (pear tree)	Pathogen of *Pyrus communis* (López et al. 2011)
*E. tasmaniensis* ET1/99	GCA_000026185.1	*Malus* sp. (apple tree)	Non-pathogen (Kube et al. 2008, 2010)
*E. typographi* M043b	GCA_000773975.1	*Ips typographus* (bark beetle)	Non-pathogen (Skrodenyte-Arbaciauskiene et al. 2012)
*E. billingiae* OSU19-1	GCF_001269445.1	*Pyrus communis* (pear tree)	Non-pathogen (Klein et al. 2015)
*E. billingiae* Eb661	GCA_000196615.1	*Malus* sp. (apple tree)	Non-pathogen (Kube et al. 2008)
*E. toletana* DAPP-PG-7351	GCA_000336255.1	*Olea* sp. (olive tree)	Pathogen associated^d^ of *Olea* sp. (Passos da Silva et al. 2013)
*Erwinia teleogrylli* SCU-B244	GCF_001484765.1	*Teleogryllus occipitalis* (mole cricket)	Non-pathogen (Liu et al. 2016)
*Erwinia* sp. 9145	GCA_000745075.1	Facultative endohyphal bacterium	Non-pathogen (Baltrus et al. 2017)
*E. oleae* DAPP-PG531	GCA_000770305.1	*Olea europaea* (olive tree)	Non-pathogen (Moretti et al. 2011, 2014)
*E. tracheiphila* BuffGH	GCA_000975275.1	*Cucurbita pepo* ssp. *Texana* (squash plant)	Pathogen of Cucurbitaceae (Shapiro et al. 2016)
*E. tracheiphila* PSU-1	GCA_000404125.1	*Cucurbita pepo* ssp. *Texana* (squash plant)	Pathogen of Cucurbitaceae (Shapiro et al. 2016)
*E. mallotivora* BT-MARDI	GCA_000590885.1	*Carica* sp. (papaya tree)	Pathogen of *Carica* sp. (Redzuan et al. 2014)
*E. persicina* NBRC-102418	GCA_001571305.1	*Piezodorus guildinii* (guts of redbanded stink bug) and Leguminosae (legume plants)	Pathogen of Leguminosae (González et al. 2007; Zhang and Nan 2014)
*Erwinia* sp. ErVv1	GCA_900068895.1	*Vitis vinifera* (grapevine)	Non-pathogen (Lopez-Fernandez et al. 2015)
*Erwinia* sp. EM595	GCA_001517405.1	*Malus* sp. (pome fruit trees)	Non-pathogen (Rezzonico et al. 2016)
*E. dacicola* Erw SC	GCA_001689725.1	*Bactrocera oleae* (olive fruit fly)	Non-pathogen (Blow et al. 2016; Estes et al. 2009)
*E. dacicola* IL	GCA_001756855.1	*Bactrocera oleae* (olive fruit fly)	Non-pathogen^e^
*Erwinia* sp. Leaf53	GCA_001422605.1	*Arabidopsis thaliana*	Non-pathogen (Bai et al. 2015)
*E. iniecta* B149	GCA_001267545.1	*Diuraphis noxia* (wheat aphid)	Non-pathogen (Campillo et al. 2015)
*E. iniecta* B120	GCA_001267535.1	*Diuraphis noxia* (wheat aphid)	Non-pathogen (Campillo et al. 2015)

^a^Nomenclature that follows Potter et al., Plant Syst. Evol., 2007 (Potter et al. 2007). However, some authors define the subfamily as Amygdaloideae

^b^No reference available

^c^Information derived from https://www.ncbi.nlm.nih.gov/biosample/SAMD00016891/ on February the 15th 2017

^d^Found on olive knots caused by the plant bacterium *Pseudomonas savastanoi* pv. *savastanoi*. The presence of *E. toletana* is correlated with the virulence of the disease suggesting a possible interactions with *P. savastanoi* pv. *Savastanoi*

^e^Here we assume that this strain is non-pathogenic based on *E. dacicola* Erw SC

*E. amylovora CFBP1430 is the reference genome where all the DNA gene sequences were extracted

^#^These strains are Rosaceae-infecting

## Results and discussion

Herein, we supply an overview on the conservation of genes important for the pathogenicity of *E. amylovora* among different *amylovora* strains and other *Erwinia* strains with deposited genomes. In Fig. 1, a heatmap shows the absence (red, <80% coverage), or presence (green, ≥80% coverage and ≥75% identity; yellow, ≥80% coverage and <75% identity) of a specific gene (bottom) within a certain *Erwinia* strain (right), and also the hierarchical relationship between the strains and the analysis outcome (left). Information about the analyzed strains is reported in Table 1, where the habitat/host and the relative plant pathogenicity are specified.

### General considerations

It is evident from the heatmap that there is a distinct separation between the group of Rosaceae-infecting strains (upper half of the figure) and the other strains. *E. tasmaniensis* ET1/99 is epiphytic and not pathogenic to plants and marks the boundary between the two groups (a wider discussion on this strain can be found below in a dedicated paragraph). The separation indicates that the genes involved in the Rosaceae-infecting strains are mostly not present, or present with a low sequence identity, in the strains not pathogenic to Rosaceae. This observation suggests that the proteins reported to be important for *Erwinia amylovora* pathogenicity are very specific to the fire blight development in Rosaceae.

### *Erwinia amylovora* strains

Most of the analyzed *E. amylovora* strains look identical to each other. However, the *Rubus*-infecting strains *E. amylovora* Ea644 and MR1 make an exception.

First, our results show that these strains lack of the *srlB* gene. The *srlB* gene is part of the sorbitol operon and codifies for a protein (SrlB) responsible for sorbitol phosphorylation during translocation into the cell (Aldridge et al. 1997). Sorbitol phosphorylation by SrlB is necessary for its internalization so that it can be exploited in the biosynthesis pathway of the exopolysaccharide (EPS) amylovoran, which is the main protective biofilm component during infection (Aldridge et al. 1997; Langlotz et al. 2011). Interestingly, unless Spiraeoideae, the *Rubus* plants (e.g., raspberries and blackberries) contain little to no sorbitol (Lee 2015; Wallaart 1980). It has been demonstrated for five tested strains of *E. amylovora* that the pathogen is able to infect apple plants with the same severity independently of sorbitol concentration (Duffy and Dandekar 2007). Moreover, it has been shown that the inability of the cells to use the sorbitol in apple shoots prevented efficient colonization of host plant tissue (Aldridge et al. 1997). Therefore, the sorbitol operon confers the ability to cope with and take advantage of the high sorbitol concentrations present inside Spiraeoideae. Consequently, the *Rubus*-infecting strains are not able to deal with one of the main carbohydrate source (i.e., sorbitol) in Spiraeoideae, precluding their ability to infect these hosts.

Second, the *rlsA* gene is absent in *E. amylovora* MR1 and has <75% identity in *E. amylovora* Ea644 when compared to the reference gene of *E. amylovora* CFBP1430. The *rlsA* product is a regulator of levan production (Zhang and Geider 1999). Levan is required for the formation of a protective biofilm and its misregulation leads to impaired infectivity in apple (Koczan et al. 2009). Our observations rise the hypothesis about the inability of *E. amylovora* Ea644 and MR1 to infect Spiraeoideae.

Furthermore, Rezzonico et al. (2012) found differences within the lipopolysaccharide (LPS) gene cluster between a *Rubus*- and a Spiraeoideae-infecting strains of *E amylovora* and they suggested that the LPS gene cluster may be used as a molecular marker to distinguish between *Rubus*- and Spiraeoideae-infecting strains of *E. amylovora* (Rezzonico et al. 2012). Herein, we suggest that also the differences in the *srlB* and *rlsA* genes loci may be used together with the analysis of the LPS gene cluster to distinguish between *Rubus*- and Spiraeoideae-infecting strains.

### *Erwinia pyrifoliae* Ep1/96, DSM-12163 and *Erwinia* sp. Ejp617

*Erwinia pyrifoliae* Ep1/96 and DSM-12163 are pathogens of *Pyrus pyrifoliae* and responsible of the Asian pear shoot blight (Geider et al. 2009; Park et al. 2011). The main difference with *E. amylovora* is that these strains have no levansucrase gene *lsc3* and no PrtA metalloprotease type 1 secretion pathway genes *prtDEF*. It was shown that *E. amylovora* Δ*lsc3* mutant cells were not detected in the xylem vessels of apple trees and were reduced in moving through apple shoots (Koczan et al. 2009). In fact, the levansucrase allows *E. amylovora* to cope with the high level of sucrose present in the Rosaceous plants as principal storage and transport carbohydrate together with sorbitol (Bogs and Geider 2000; Geier and Geider 1993; Gross et al. 1992). While, the missing PrtA protease secretion was reported to reduce colonization of *E. amylovora* in the parenchyma of apple leaves (Zhang et al. 1999). Therefore, the lack of *lsc3* and *prtDEF* genes may be correlated with the limited host-range and decreased virulence of *E. pyrifoliae* respect the fire blight-causing bacteria. The DSM-12163 strain is also missing the cysteine protease effector-gene *avrRpt2*, which is believed to have been acquired by *E. amylovora* after the separation from *E. pyrifoliae* species (Zhao et al. 2006). However, we found that *E. pyrifoliae* Ep1/96 harbors the *avrRpt2* gene, indicating that the hypothesis about its acquisition should be still considered controversial.

*Erwinia* sp. Ejp617 is a pathogen of *Pyrus pyrifolia* and causes the bacterial shoot blight of pear (BSBP) (Park et al. 2011). It shows a heatmap profile similar to *E. pyrifoliae* DSM-12163, but it also lacks of the *eop2* and the *hsvC* genes. *Eop2* codifies for a type 3 secreted effector/helper protein bearing a pectate lyase domain (Asselin et al. 2006), while the missing *hsvC* (hrp-associated systemic virulence protein C) gene codifies for a carboxylate lyase required for full virulence in apple (Oh et al. 2005). These observations are consistent with the fact that *Erwinia* sp. Ejp617 is not able to cause fire blight and indicate that the *eop2*, *hsvC*, *lsc3* and *avrRpt2* genes are not necessary to infect *Pyrus* shoots, but discriminating when it comes to spread the infection to the whole plant.

### *Erwinia piriflorinigrans* CFBP-5888

*Erwinia piriflorinigrans* is a *Pyrus communis* pathogen whose infection is limited to the blossoms (López et al. 2011; Roselló et al. 2006). Infected blossoms are similar in appearance to those affected by the fire blight caused by *Erwinia amylovora*. The *E. piriflorinigrans* CFBP-5888 strain is lacking of a number of genes present in *E. amylovora*.

The entire sorbitol operon is missing and can be related to its inability to infect the internal part of the plant. In fact, as already mentioned, the *srl* operon is important to exploit sorbitol within Spiraeoideae (Aldridge et al. 1997). The missing *hrpY* gene product is part of an upstream 2-component system regulating the *hrp* gene cluster together with HrpX (Wei et al. 2000). The latter works as a sensor protein and HrpY works as the response regulator partner. This means that in *E. piriflorinigrans* CFBP-5888 there is an impaired regulation of the *hrp* gene cluster.

The *hsvABC* genes are missing. They are required for full virulence in apple (Oh et al. 2005). Then, the missing *hrpW* gene codifies for a pectate lyase-like harpin protein and thereby is an effector of infection (Gaudriault et al. 1998). The missing *avrRpt2* gene, as already mentioned, codifies for a cysteine protease T3SS effector important for virulence in apple trees (Zhao et al. 2006). Moreover, *E. piriflorinigrans* CFBP-5888 lacks the *prtABCDE* gene cluster. As previously discussed, the products of this cluster form a type 1 secretion system where the PrtA protein is a secreted metalloprotease demonstrated to influence the ability to colonize the parenchyma of apple leaves (Zhang et al. 1999). The missing *eop1*-*2* genes encode for type 3 effector proteins, whose role remains unknown (Zhao and Qi 2011). Based on sequence divergence among *Rubus* or Spiraeoideae-infecting strains and mutational studies, Asselin et al. suggested that the Eop1/YopJ protein is a host-range-limiting factor that could act as a host specificity determinant towards, either *Rubus*, or Spiraeoideae (Asselin et al. 2011). In fact, sequencing of the *orfA*-*eop1* regions of several strains of *E. amylovora* revealed that different forms of *eop1* are conserved among strains with similar host ranges. In addition, mutational experiments showed that *eop1* can otherwise influence virulence when heterologously expressed in *Rubus* or Spiraeoideae based on the strain it comes from. However, a transposon insertion mutant in the *eop1* gene of the Spiraeoideae-infecting strain *E. amylovora* Ea273/ATCC-49946 (Ea273 eop1::Tn) caused symptoms similar to those of the wild-type strain. Therefore, it is plausible that the lack of *eop1* has no effect on the infectivity of *E. piriflorinigrans* CFBP-5888. The missing *edcE* gene codifies for a diguanylate cyclase involved in the production of c-di-GMP, which positively regulates the secretion of amylovoran, leading to increase biofilm formation and negatively regulating flagellar swimming motility (Edmunds et al. 2013). The missing *rlsB* gene product is a positive regulator of levan synthesis and its absence may downregulate levansucrase expression and suppress levan production (Du and Geider 2002). The missing *amsD* gene codifies for a glycosyltransferase part of the amylovoran biosynthesis machinery. The AmsD protein attaches the second galactose residue to the growing repeating unit of the amylovoran precursor (Langlotz et al. 2011). Overall, the lack of both *edcE*, *rlsB* and *amsD* can lead to a lower or impaired EPS production in the *E. piriflorinigrans* CFBP-5888 strain that could correlate to the inability of this species to colonize the phloem. The missing *ycfA* gene codifies for a protein crucial for the 6-thioguanine (6TG) biosynthesis, which is a cytotoxin released from *E. amylovora* (Coyne et al. 2013). The *ΔyfcA* mutant revealed the crucial role of 6TG and, therefore, of YfcA in the development of the fire blight disease in apple plants.

Overall, our results on the *E. piriflorinigrans* CFBP-5888 strain suggest that the lack of the described genes may have drifted the pathogenicity towards *Pyrus* blossoms infections.

Intriguingly, the common missing genes among the *Pyrus-*infecting strains are restricted to the metalloprotease PrtA secretion system that, being an important player in the colonization of the parenchyma of apple, might represent one of the principal determinants in host specificity. On the other hand, we showed that the missing genes in *E. piriflorinigrans* CFBP-5888 are not necessary to infect blossoms.

### *Erwinia tasmaniensis* ET1/99 strain

*Erwinia tasmaniensis* ET1/99 strain marks the border between the Rosaceae pathogens and the other strains. It is evident that *E. tasmaniensis* ET1/99 presents many similarities to the pear tree pathogen *E. piriflorinigrans* CFBP-5888. However, the *ycfA*, *hrpW* and *hrpY* genes are missing in *E. piriflorinigrans* CFBP-5888 and present in *E. tasmaniensis* ET1/99. Conversely, several genes that are present in the *piriflorinigrans* strain are missing in the *tasmaniensis* strain: *dspF*, *hrpA*, *hrpK, amsE*, *amsK* and *edcC*. Besides, the *dspE* gene in *E. tasmaniensis* ET1/99 has a <75% sequence identity compared to the reference sequence. Hence, the further absences of *E. tasmaniensis* ET1/99 may correlate to its inability to be infective. The disease specific (*dsp*) Hrp-associated pathogenicity-avirulence proteins DspE/A and DspF/B are among the principal effector in the fire blight disease and required for pathogenesis in Maloideae (Bogdanove et al. 1998; Gaudriault et al. 1997). The *hrpA* gene is part of the *hrp* operon, which is required for secretion of harpins and/or effectors and predicted to be an ATP-dependent helicase (Choi et al. 2013; Kim et al. 1997). The *hrpK* gene is part of the *E. amylovora* pathogenicity island. The codified protein HrpK is secreted and was suggested to be a translocator able to create channels in the plasma membrane of plant cells, although its actual function in fire blight remains to be determined (Oh et al. 2005). The *amsE* and *amsK* genes are part of the amylovoran-synthesis operon. The encoded AmsE and AmsK proteins are glucoside transferases that transfer the third and the last galactose residues, respectively, on the amylovoran precursor (Langlotz et al. 2011). Hence, their importance in proper amylovoran production and thereafter biofilm formations are clear. Eventually, as the *edcE* gene, the missing *ecdC* gene codifies for a diguanylate cyclase that positively regulates the secretion of amylovoran. Thereafter, the lack of genes whose products are considered to be critical for Rosaceae infection, well explain why the *E. tasmaniensis* ET1/99 strain is non-pathogenic respect the *E. piriflorinigrans* CFBP-5888 strain.

### Non-Rosaceae pathogens and non-pathogens

Four strains, *E. tracheiphila* BuffGH, *E. tracheiphila* PSU-1, *E. mallotivora* BT-MARDI and *E. persicina* NBRC-102418, are reported to be pathogens of Cucurbitaceae (the first two), papaya tree and Leguminosae, respectively. However, they show no evident difference from the non-pathogenic strains in respect the heatmap outcome, again suggesting that most of the analyzed genes are not necessary for general pathogenesis, but they are host-specific. Only three genes (*relA*, *dskA*, *csrA*) have been found in most of the analyzed strains, pointing towards an important role besides pathogenesis. The *relA* gene codifies for a ribosome-associated protein engaged in the synthesis of ppGpp (Zhang and Geider 1999) and is present in all analyzed strains of *Erwinia*. The ppGpp interacts with the RNA polymerase (RNAP) to inhibit, or activate genes. The *dskA* gene product modulates the ppGpp-RNAP interaction enhancing the ppGpp effect (Ancona et al. 2015). The *dksA* gene is missing only in *Erwinia* sp. Leaf53. The *csrA* gene product is a post-transcriptional regulator of motility, amylovoran production, T3SS and virulence (Ancona et al. 2016). The *csrA* is not present in *Erwinia* sp. SCU-B244.

## Conclusion

The *Erwinia amylovora* species can be divided into two host-specific groupings: the Spiraeoideae-infecting (*e.g.*, *Malus*, *Pyrus*, *Crataegus*, *Sorbus*) and the *Rubus*-infecting strains such as *E. amylovora* Ea644 and MR1 (Mann et al. 2013). We suggest that the difference in host specificity could be correlated with the lack in the *Rubus*-infecting bacteria of a complete sorbitol operon. Thus, restricting the infectivity of *E. amylovora* Ea644 and MR1 to *Rubus* plants, which have little to no sugar alcohols, respect to other Rosaceae such as *Malus* and *Pyrus* (Lee 2015). Then, we suggested that the analysis of the *srlB* and *rlsA* loci may be used together with the analysis of the LPS gene cluster to distinguish between *Rubus*- and Spiraeoideae-infecting strains.

We hint that the host specificity of the *Pyrus*-infecting strains may be guided by the lack of genes involved in biofilm formation and virulence in apple. Intriguingly, all the *Pyrus*-infecting strains are impaired in the PrtA secretion system and, therefore, it would be interesting to investigate the virulence variation of *E. amylovora* apple infecting strains when mutated in the *prt* operon. Then, under the light of our observations, we advise that the hypothesis of the *avrRpt2* acquisition after the phylogenetic separation of *E. amylovora* from *E. pyrifoliae* should be reconsidered. We discovered that the *eop2*, *hsvC*, *lsc3* and *avrRpt2* genes are not necessary to infect *Pyrus* shoots, but they are required for the whole plant infection. We proposed that the lack of both *edcE*, *rlsB* and *amsD* in *E. piriflorinigrans* CFBP-5888 might have drifted the pathogenicity towards *Pyrus* blossoms infections. Then, we suggest that the PrtA type 1 secretion system might represent one of the principal determinants in the host specificity towards the pear plants., Considering that the virulence of the *Pyrus-*infecting strains is lower than the virulence of the fire blight-causing bacteria (Smits et al. 2011; Zhao et al. 2006), we propose that their pathogenicity towards pear trees could be addressed to the loss of ability to infect apple trees due to the described gene loss, rather than to a spontaneous evolutionary drift towards a different host. However, more studies are needed to clarify this interesting issue.

Our observations on *E. tasmaniensis* ET1/99, which is an epiphytic bacterium marking the boundary with the Rosaceae-infecting and non-infecting bacteria, hint that the lack of genes whose products are considered to be crucial for Rosaceae infection, well explain why the *E. tasmaniensis* ET1/99 strain is non-pathogenic.

The most conserved genes among all the considered *Erwinia* strains are *relA*, *dksA* and *csrA/rsmA*. However, they are not always present, indicating that they are not necessary for survival, but important in *Erwinia amylovora* pathogenicity for their general role in regulating transcription and translation.

In conclusion, our results indicate that most of the analyzed genes are not necessary for general pathogenesis, but they are specific for the infection of Rosaceae plants. Future studies should aim to clarify the correlations highlighted within the presented work to increase our knowledge about host specificity and pathogenesis within the *Erwinia* genus.
